# Convolutional neural network for the diagnosis of early gastric cancer based on magnifying narrow band imaging

**DOI:** 10.1007/s10120-019-00992-2

**Published:** 2019-07-22

**Authors:** Lan Li, Yishu Chen, Zhe Shen, Xuequn Zhang, Jianzhong Sang, Yong Ding, Xiaoyun Yang, Jun Li, Ming Chen, Chaohui Jin, Chunlei Chen, Chaohui Yu

**Affiliations:** 1 Department of Gastroenterology, The First Affiliated Hospital, College of Medicine, Zhejiang University, 79 Qingchun Road, Hangzhou, 310003 China; 2 Department of Gastroenterology, Yuyao People’s Hospital, Yuyao, China; 3 Department of Gastroenterology, The Affiliated Hospital of School of Medicine of Ningbo University, Ningbo, China; 4 Department of Gastroenterology, Jinhua Hospital, Zhejiang University School of Medicine, Jinhua, China; 5 Department of Pathology, The First Affiliated Hospital, College of Medicine, Zhejiang University, Hangzhou, China; 6 Hithink RoyalFlush Information Network Co., Ltd, Hangzhou, China; 7 State Key Laboratory for Diagnosis and Treatment of Infectious Disease, Collaborative Innovation Center for Diagnosis and Treatment of Infectious Diseases, The First Affiliated Hospital, College of Medicine, Zhejiang University, Hangzhou, China

**Keywords:** Magnifying endoscopy, Narrow band imaging, Convolutional neural network, Early gastric cancer

## Abstract

**Background:**

Magnifying endoscopy with narrow band imaging (M-NBI) has been applied to examine early gastric cancer by observing microvascular architecture and microsurface structure of gastric mucosal lesions. However, the diagnostic efficacy of non-experts in differentiating early gastric cancer from non-cancerous lesions by M-NBI remained far from satisfactory. In this study, we developed a new system based on convolutional neural network (CNN) to analyze gastric mucosal lesions observed by M-NBI.

**Methods:**

A total of 386 images of non-cancerous lesions and 1702 images of early gastric cancer were collected to train and establish a CNN model (Inception-v3). Then a total of 341 endoscopic images (171 non-cancerous lesions and 170 early gastric cancer) were selected to evaluate the diagnostic capabilities of CNN and endoscopists. Primary outcome measures included diagnostic accuracy, sensitivity, specificity, positive predictive value, and negative predictive value.

**Results:**

The sensitivity, specificity, and accuracy of CNN system in the diagnosis of early gastric cancer were 91.18%, 90.64%, and 90.91%, respectively. No significant difference was spotted in the specificity and accuracy of diagnosis between CNN and experts. However, the diagnostic sensitivity of CNN was significantly higher than that of the experts. Furthermore, the diagnostic sensitivity, specificity and accuracy of CNN were significantly higher than those of the non-experts.

**Conclusions:**

Our CNN system showed high accuracy, sensitivity and specificity in the diagnosis of early gastric cancer. It is anticipated that more progress will be made in optimization of the CNN diagnostic system and further development of artificial intelligence in the medical field.

## Introduction

Gastric cancer is one of the most prevalent tumors and the third leading cause of cancer-related death worldwide [1, 2]. Most gastric mucosal lesions develop in a stepwise manner from atrophy, intestinal metaplasia, low-grade intraepithelial neoplasia, high-grade intraepithelial neoplasia, and finally to gastric cancer [3, 4]. An improvement in the accuracy of endoscopic diagnosis for early gastric cancer and precancerous lesions will be substantially helpful in reducing the incidence and mortality of gastric cancer. Moreover, if the lesions can be detected before further progression to invasive cancer, most of the gastric mucosal lesions can be removed by endoscopic resection and the patients will show a significant improvement in health-related quality of life [5]. Therefore, it is of great importance to work to achieve a better diagnostic accuracy of gastric mucosal lesions for the prevention and treatment of gastric cancer.

Magnifying endoscopy with narrow band imaging (M-NBI) has been used to examine glandular epithelium in the stomach by observing microvascular architecture and microsurface structure, which enjoys a significantly better accuracy than common white light endoscopes [6–9]. Experts recommended an algorithm called magnifying endoscopy simple diagnostic algorithm for early gastric cancer (MESDA-G) to distinguish between non-cancerous lesions and early gastric cancers [10]. Based on this algorithm, several studies have reported that the sensitivity of M-NBI in the diagnosis of early gastric cancer ranged from 85.7 to 97.3% and the specificity from 84.4 to 96.8% [11–13]. However, the diagnostic efficacy of non-experts in differentiating early gastric cancer from non-cancerous lesions by M-NBI was usually disappointing [14–16].

To overcome these shortcomings, artificial intelligence was introduced to improve medical diagnosis [17, 18]. At present, deep learning in the field of artificial intelligence has completely broken the bottleneck of traditional machine learning, so that medical tasks such as image classification can be better performed, and the application of artificial intelligence in medical practice can be further explored [19, 20]. Convolutional neural network (CNN) is one of the most representative network models in the field of deep learning. As a research hotspot in current image processing, CNN has achieved tremendous success and wide application in image recognition and classification [21, 22]. Therefore, we applied CNN to endoscopic diagnosis in an attempt to further improve diagnostic efficacy of early gastric cancer. In the present study, we developed a novel system based on CNN to analyze gastric mucosal lesions observed by M-NBI.

## Materials and methods

### Data preparation

**Fig. 1:**
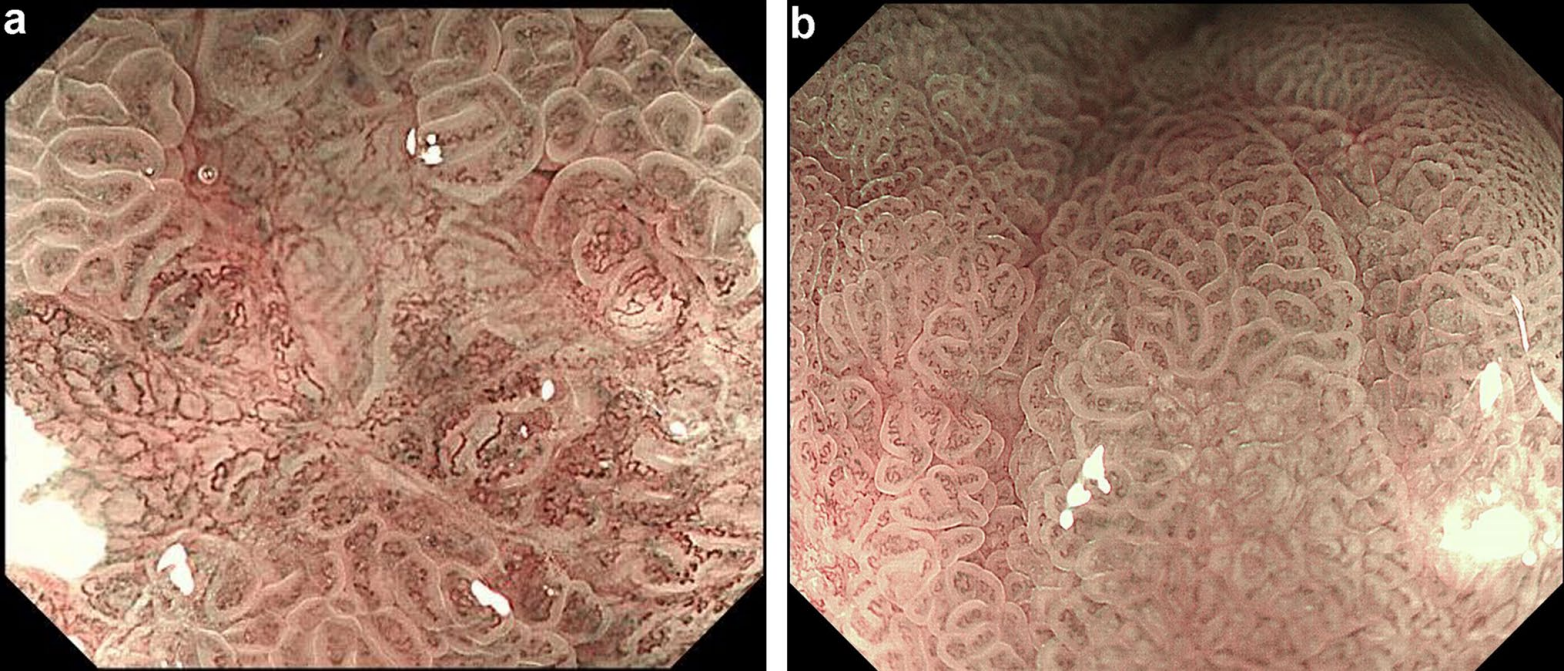
Representative M-NBI images of gastric mucosal lesions. **a** Image was diagnosed as early gastric cancer; **b** image was diagnosed as non-cancerous lesion

The study was approved by medical ethics committee of each study center. Written informed consent was obtained from each patient. An upper gastrointestinal endoscope (GIF-H260Z or GIF-H290Z, Olympus, Tokyo, Japan) with magnifying and NBI function was used to observe gastric mucosal lesions. The structure enhancement function was usually preset as B mode levels 4, 6, and 8 with NBI color mode 1, but in actual practice endoscopists usually adopted level 8 structure enhancement with NBI. Two endoscopists who have more than ten years of work experience retrospectively diagnosed gastric mucosal lesions using M-NBI. The lesions were examined at maximal magnification during NBI observation. The lesions were classified as non-cancerous lesions and early gastric cancer according to vessels plus surface classification system and MESDA-G (Fig. 1) [6, 7, 10]. Meanwhile, two pathologists performed histological evaluation of the corresponding lesions according to the revised Vienna classification of gastrointestinal epithelial neoplasia [23]. Lesions of Category 4 (mucosal high-grade neoplasia) and category 5 (submucosal invasion by carcinoma) were diagnosed as early gastric cancer, whereas category 1 (negative for neoplasia), category 2 (indefinite for neoplasia) and category 3 (mucosal low-grade neoplasia) lesions were diagnosed as non-cancerous lesions. The pathologists made the diagnoses of category 4 and 5 from the resected specimens and made the diagnoses of category 1 and 2 from the biopsy specimens, while category 3 lesions were diagnosed partly based on the biopsy specimens and partly based on the resected specimens. When the endoscopic diagnosis showed inconsistence with the pathological finding, the pathologists and the endoscopists would make reassessment and discussion to reach a consensus.

A total of 386 images of non-cancerous lesions and 1702 images of early gastric cancer from four hospitals in four areas of Zhejiang province were collected to train and establish a CNN model. No significant age and gender differences were observed between patients in the non-cancerous group and the early cancer group. Polypoid lesions (type 0-I), ulcerated lesions (type 0-III), advanced gastric cancer and low-quality images, such as presence of mucus on mucosal surface or severe mucosal bleeding, were excluded during the process of modeling.

Considering that the imbalance between the two types of images might affect the convergence during the training phase and lead to poor generalization ability and model over-fit [24], we increased the sample size by following such image processing methods as image rotate transform, image mirror transform, image cut, image brightness variations, image blur, and up-sampling, which could balance the number of different types of samples [25]. A total of 10,000 images of non-cancer and 10,000 images of early gastric cancer were enrolled to train our CNN model.

## Training algorithm

**Fig. 2:**
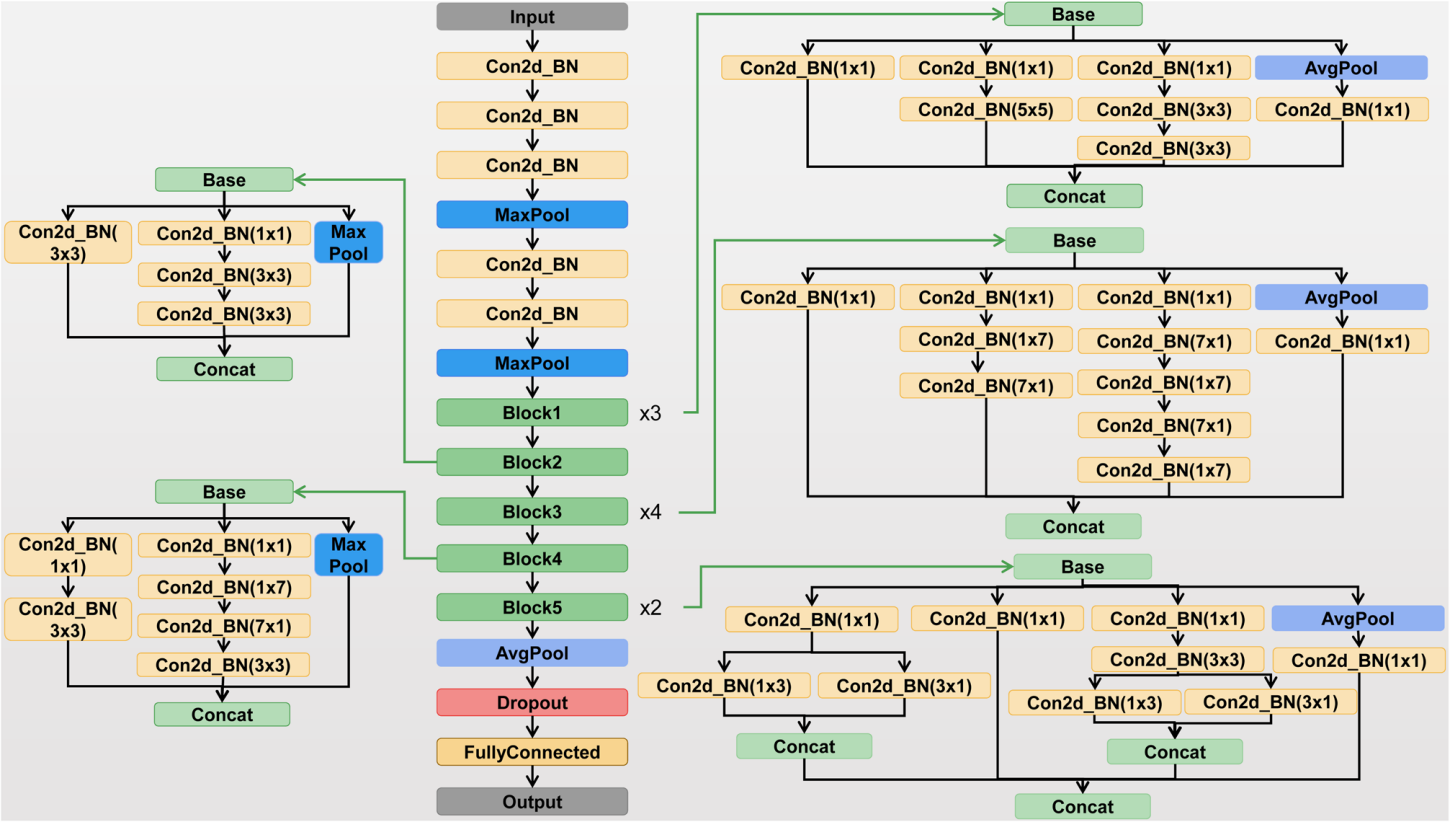
Inception v3 model architecture

As one of the classical models in CNN, Inception-v3 model consists of 11 Inception modules. The Inception module used filters of different sizes to process the input characteristics, and then the last layer of the modules combined the results of different filters. An important improvement of Inception-v3 was that it factorized convolutions of a large filter size into smaller ones, which increased the computational efficiency, deepened the network and increased the non-linearity of the network [26]. The architecture of Inception v3 model is shown in Fig. 2.

Keras deep learning framework, a highly modular neural network library featuring its simple use and fast training, was used to train Inception-v3 model. The model training process included the following steps: (1) create the model and load the public pretrained inception-v3 model to initialize the model weights; (2) remove the top layer and replace the full-connected layer with the output value of 2 (one type is early cancer and the other is non-cancer) as the new top layer; (3) fine-tuning; (4) accelerate the convergence of the model.

During the model training, the input size of the model was changed from 299 × 299 pixels to 512 × 512 pixels, to extract more characteristic information from the input pictures and improve the accuracy of the model.

### Testing algorithm

The two experienced endoscopists mentioned above observed and photographed the M-NBI images from patients with gastric mucosal lesions collected from four hospitals between January 2017 and March 2018. Each lesion was pathologically examined by the two pathologists mentioned above according to the revised Vienna classification of gastrointestinal epithelial neoplasia. The protocol for pathological diagnosis has been described in the data preparation phase. Exclusion criteria included polypoid lesions (type 0-I), ulcerated lesions (type 0-III), advanced gastric cancer and low-quality images. In the light of previous studies [11–13, 27–29], the expected value of sensitivity was 83% for the diagnosis of early gastric cancer using M-NBI and the expected value of specificity was 84%. With an alpha level of 0.05 and a permissible error of 0.06, the minimum required sample size for early gastric cancer and non-cancerous group was 151 and 143, respectively. In our study, we prospectively enrolled a total of 341 endoscopic images (170 early gastric cancer and 171 non-cancerous lesions) to evaluate and compare the diagnostic capabilities of CNN and endoscopists. The image-quality criteria for testing accorded with that for training.

Two experts and two non-experts who were unaware of the pathological findings processed these testing images. The experts, with more than 10 years of work experience, complete an average of more than 1000 cases of gastroscopy each year with an early gastric cancer detection rate > 5%. The non-experts have 3 years of work experience and complete more than 120 cases of gastroscopy each year. The testing images were displayed on the laptop in a random order. Both experts and non-experts were asked to divide the images into non-cancerous lesion and early gastric cancer.

### Statistical analysis

Primary outcome measures included diagnostic accuracy, sensitivity, specificity, positive predictive value (PPV), and negative predictive value (NPV). The two-sided McNemar test was used to compare the accuracy, sensitivity, and specificity between endoscopists and CNN. The Chi-square test was used to compare the PPV and NPV between endoscopists and CNN. Cohen’s kappa coefficient was calculated to evaluate interobserver agreement. A value of *P* < 0.05 was considered as statistically significant.

## Results

From January 2017 to March 2018, we prospectively enrolled 171 cases of non-cancerous lesion and 170 cases of early gastric cancer to test the diagnostic capability of CNN (Table 1). Of the 171 cases of non-cancerous lesions, 45 were located at the fundus, 56 at the corpus, 10 at the angle, and 60 at the antrum. According to the revised Vienna classification, 104 cases were negative for neoplasia (21 normal epithelium, 35 hyperplastic epithelium, and 48 intestinalized epithelium with or without atrophy), and 67 cases were low-grade neoplasia. Of the 170 cases of early gastric cancer, 42 were located at the fundus, 49 at the corpus, 13 at the angle, and 66 at the antrum. According to the revised Vienna classification, 58 cases were high-grade adenoma/dysplasia, 59 cases were carcinoma in situ, 1 case was suspicious of invasive carcinoma, 49 cases were intramucosal carcinoma, and 3 cases were submucosal invasion by carcinoma. According to Paris classification of morphology, 39 cases were type 0-IIa, 30 cases were type 0-IIb, 36 cases were type 0-IIc, 57 cases were type 0-IIa + IIc, and 8 cases were type 0-IIc + IIa.

**Table 1:** Clinicopathologic characteristics of gastric mucosal lesions in the test set

	Early gastric cancer (*n* = 170)	Non-cancerous lesion (*n* = 171)
Location		
Fundus	42	45
Corpus	49	56
Angle	13	10
Antrum	66	60
Morphology		
0-IIa	39	
0-IIb	30	
0-IIc	36	
0-IIa + IIc	57	
0-IIc + IIa	8	
Pathology		
Negative for neoplasia		104
Mucosal low-grade neoplasia		67
High-grade adenoma/dysplasia	58	
Carcinoma in situ	59	
Suspicious for invasive carcinoma	1	
Intramucosal carcinoma	49	
Submucosal invasion by carcinoma	3	

The performance of diagnosis by CNN and endoscopists are shown in Table 2. Among 170 cases of early gastric cancer, 155 cases were correctly classified by CNN and the diagnostic sensitivity was 91.18%. Among 171 cases of non-cancerous lesions, 155 cases were properly classified by CNN and the diagnostic specificity was 90.64%. The accuracy of CNN in the diagnosis of early gastric cancer was 90.91%.

**Table 2:** Diagnostic performance of CNN versus endoscopists in differentiating early gastric cancer and non-cancerous lesion

	Expert 1	Expert 2	Non-expert 1	Non-expert 2	CNN
Sensitivity	78.24*	81.18*	77.65*	74.12*	91.18
Specificity	94.74	93.57	61.99*	73.10*	90.64
PPV	93.66	92.62	67.01*	73.26*	90.64
NPV	81.41*	83.33*	73.61*	73.96*	91.18
Accuracy	86.51	87.39	69.79*	73.61*	90.91

^*^Significant difference compared with CNN

There was no significant difference in the specificity and accuracy of diagnosis between CNN and experts. However, the diagnostic sensitivity of CNN was significantly higher than that of the experts (all *P* < 0.01). Furthermore, the diagnostic sensitivity, specificity, and accuracy of CNN were significantly higher than those of the non-experts (all *P* < 0.001). The interobserver agreement of the two experts was good (kappa scores of 0.766), and the interobserver agreement of the two non-experts was relatively poor (kappa score of 0.289). The interobserver agreement between CNN and experts (kappa scores of 0.719 and 0.654) was better than that between experts and non-experts (kappa scores of 0.466 and 0.331).

## Discussion

In the present study, we retrospectively reviewed 386 images of non-cancerous lesions and 1702 images of early gastric cancer for CNN training. After the enlargement of sample size by image preprocessing, CNN system was established based on 20,000 training images. Then we prospectively enrolled 171 images of non-cancer and 171 images of early gastric cancer to test and evaluate the diagnostic capability of CNN.

Given the fact that the disease-free survival and overall survival of patients with early gastric cancer were significantly higher than those of patients with advanced gastric cancer [30, 31], the diagnosis of early gastric cancer is very important for selecting subsequent treatment programs and improving the quality of life of patients. The endoscopic appearance of early gastric cancer is usually very subtle, making it difficult to differentiate early gastric cancer from non-cancerous lesions. As a result, the identification of early gastric cancer depends largely on the personal judgement of endoscopists. Therefore, it is essential to develop new ways to improve optical diagnosis that differentiates between non-cancerous lesion and early gastric cancer.

M-NBI system is currently one of the most powerful tools for evaluating the microvessels and microstructure of the gastrointestinal mucosa. The sensitivity and specificity of M-NBI images in the diagnosis of early gastric cancer were significantly higher than those of conventional white light images [13, 27, 32, 33]. However, the non-experts often exhibited poor sensitivity and specificity in diagnosing early gastric cancer with M-NBI [14–16]. A potential solution to improve optical diagnosis is to apply CNN to M-NBI diagnosis. CNN forms abstract high-level representation features by combining low-level features, thereby discovering the distributed feature representations of data. CNN is more similar to biological neural network owing to its local links and weight sharing, which not only reduce the complexity of network mode and the number of weights, but also make the model more adaptable. At the beginning, CNN needed a few hours to complete the 10-epochs training process to generate the identification system. Once the training process was completed, the identification system could be used repeatedly. The core identification system enjoys the merit of good adaptability as it can be used on multiple platforms for real-time analysis of the JPEG images captured by M-NBI. In addition, the magnifying and clear images provided by M-NBI can improve the speed and accuracy of CNN diagnostic system compared with images provided by conventional endoscopy.

In the present study, each training image possibly enrolled was first judged by experienced endoscopists and confirmed pathologically, during which only M-NBI images with appropriate magnification and typical manifestation were selected for CNN model learning. The testing results showed that CNN diagnostic system achieved better sensitivity than experts and non-experts, and there was no significant difference with respect to specificity between CNN and experts. Moreover, the diagnostic concordance between CNN and the two experts was 82.70% and 85.92%, respectively. Therefore, CNN performed quite well in detecting NBI features of early gastric cancer or non-cancerous lesions and showed the potential to provide diagnostic assistance for endoscopic physicians in the future practice, especially for those with insufficient experience.

We detected good interobserver agreement between the two experts and a moderate agreement between CNN and experts, while the non-experts displayed poor interobserver agreement. Accordingly, the interobserver agreement between CNN and non-experts was also poor. The reason lies in that the software could avoid subjectivity when processing and diagnosing NBI images, whereas an inevitable element of personal judgement does exist when it comes to endoscopists, who usually need a period of training and practice to become skilled and proficient in clinical application of M-NBI. Therefore, CNN may work as a “confirmer” or “corrector”. A second diagnosis by CNN may help reduce diagnostic errors made by endoscopists earlier and develop optimal treatment plans.

There are several limitations to this study. First, similar Japanese studies using M-NBI for early gastric cancer diagnosis achieved considerably higher sensitivity and accuracy than the present study [12, 13, 34]. We speculate that the discrepancies may be due to the different histological interpretation and nomenclature rules between Japanese pathologists and Chinese pathologists. Japanese pathologists used the revised Vienna classification combined with Japanese pathological diagnostic criteria for early gastric cancer diagnosis [35], whereas we used the Vienna classification system only. Second, considering that M-NBI is suitable for identifying benign and malignant characteristics of non-polypoid and nonexcavated lesions, type 0-I and type 0-III lesions were excluded from the present study, which restricted the application range of CNN system. Despite these limitations, the present study provided a potential and powerful CNN diagnostic system for the differential diagnosis of non-cancerous lesions and early gastric cancer.

In conclusion, a combination of CNN system and M-NBI in our study showed high accuracy, sensitivity, and specificity in the diagnosis of early gastric cancer. It is anticipated that more headway will be made in the endoscopic diagnosis of early gastric cancer by CNN and the further development of artificial intelligence in medical practice.
